# Audit to optimize antibiotics use in neonatal septicemia

**DOI:** 10.12669/pjms.38.6.5073

**Published:** 2022

**Authors:** Ayesha Usman, Maria Zamurad Khan, Maimoona Zamurad Khan, Abdullah Hussain

**Affiliations:** 1 Ayesha Usman, MBBS. Services Hospital, Lahore, Pakistan; 2 Maria Zamurad Khan, MBBS. Mayo Hospital, Lahore, Pakistan; 3 Maimoona Zamurad Khan, MBBS. Jinnah Hospital, Lahore, Pakistan. Mayo Hospital, Lahore, Pakistan; 4 Abdullah Hussain, MBBS. Services Hospital, Lahore, Pakistan

**Keywords:** Antibiotic stewardship program, Antibiotic prescription, Neonatal sepsis

## Abstract

**Objectives:**

To assess appropriate antibiotics use in neonatal sepsis and to highlight the need for developing an Antibiotic stewardship program at local levels.

**Methods:**

A clinical audit was conducted in the neonatal ward of the tertiary care hospital of Lahore for one year from May 2019 to May 2020. Reports of blood culture and drugs susceptibility were gathered from the microbiology department, and clinical records were evaluated about the choice of the antimicrobials, dosage, frequency, and clinical prognosis. The statistics were applied using SPSS software.

**Results:**

Eighty five neonates with the mean age of five days were treated in tertiary care hospital for septicemia. Every patient received more than one antibiotic empirically. The most prescribed drug combination (90.6%) was *Cefotaxime* and *Amikacin*. Optimum antibiotics dose was prescribed in only 70.2% of cases. Blood isolates showed gram-negative bacilli in 69 (81.2%) cases, gram positive cocci in 14 (16.5%) cases, two (2.3%) culture susceptibility reports showed growth of candida. Gram negative organisms were most susceptible to *Imipenem* (54%), *Piperacillin-Tazobactam* (48%) and *Gentamicin* (48%). Gram-positive organisms showed the most susceptibility to *Vancomycin* (100%), *Amikacin* (92%), and *Co-amoxiclav* (85%). *Meropenem* (39%), *Linezolid* (28%), and *Vancomycin* (27%) were the most commonly given alternate antibiotics. All the patients (n=10, 11.8%) whose culture sensitivity reporting showed susceptibility to empirical therapy survived.

**Conclusion:**

Due to poor availability of latest data about local antibacterial resistance pattern and lack of knowledge among pediatricians about latest antibiotic prescribing protocols, many inconsistencies were noted in the use of antibiotics in neonatal sepsis which resulted in a poor outcome hence, reflecting in international key health indicators (neonatal mortality rate) of country. Concerning the change in the resistance pattern of microorganisms to antimicrobials, it is high time to collect local data about antibacterial susceptibility and develop an antibiotic stewardship program to stop inappropriate use of antibiotics.

## INTRODUCTION

The first few weeks of the neonate are the most critical and vulnerable period of life when a minor ailment will result in neonatal demise if not managed optimally. There are approximately 2.4 million neonatal deaths every year worldwide with the majority occurring within the first week of life.^1^ The three significant reasons for neonatal death globally are infections (36%), pre-term delivery (28%), and birth asphyxia (23%). These infections include sepsis/pneumonia, diarrhea, and tetanus.^2^ Neonatal infections are a significant cause of neonatal mortality and morbidity causing 30-50% neonatal deaths per year in developing countries.^3^

In Pakistan, 41 neonates out of 1000 live births die every year.^4^ Neonatal sepsis shows a high incidence in developing countries like Pakistan due to poor access to antenatal care, home deliveries, and inefficient practices of infection control in healthcare facilities. Early diagnosis and appropriate treatment of septicemia are the only strategies to prevent mortality and morbidity.

In developing countries, the use of antimicrobials is mostly irrational based on guess work.^5^ Due to the poor availability of investigation facilities, physicians prescribe antibiotics on clinical suspicion, these mostly include broad-spectrum antibiotics as the causative organism, and their antimicrobial susceptibility patterns are not known to the treating physicians. Therefore, this study was undertaken to determine antibiotic resistance patterns in neonatal infections and to evaluate the use of antibiotics in neonates admitted with sepsis.

## METHODS

Data of all the neonates admitted with the suspicion of sepsis during this time was observed. Eighty five patients with clinically significant isolates recovered from blood were included in the study. The preparation of blood cultures and identification of the organism and susceptibility testing of antibiotics were performed according to standard microbiological techniques of culture & sensitivity by Clinical & Laboratory Standard Institutes (CLSI) guidelines.^6^ Culture and susceptibility data were recorded for analysis. Patient information about demographic details, bacteriology data, and the possible source of bacteremia was noted together with any significant underlying illness. Selection of the antimicrobial drug(s), route of administration, dose, and frequency of antibiotic treatment was assessed. The quality of this management was evaluated regarding the organism(s) isolated, the underlying source of infection, and the suggestions made by the hospital microbiological team. Data were also recorded about changes in treatment recommended based on Gram stain and culture and drug susceptibility results.

Approval from the institutional review board of tertiary care hospital in Lahore (Ref No. IRB/2019/237/SIMS; dated: 06-05-2019) was obtained to proceed with a prospective case series study in the pediatric ward for the period of one year from May 2019 to May 2020.

### Statistical Analysis

Statistical package for social sciences (SPSS version 21) was used for analyzing data. Mean and standard deviation was calculated for quantitative variables, while for qualitative variables percentage and frequency distribution tables were made. Diagrams (bar charts) were also used for data presentation.

## RESULTS

Analysis of Demographic characteristics showed that Male to Female ratio was 1.7:1. The mean age was 5.25 days (SD±7.027) with 47(55.3%) neonates presenting on the first day of life. Among these, 52(61.2%) were Low Birth Weight (less than 2500g), 28(32.9%) were born Preterm, while 20(23.5%) suffered Birth Asphyxia. 45(52.9%) patients were admitted for sepsis {24(28.2%) patients presenting with Early Onset Sepsis while 21(24.7%) with Late-Onset Sepsis}, 20(23.5%) had pneumonia while 10(11.8%) patients had Meningoencephalitis as clinical diagnosis. However, 10(11.8%) patients were being treated for non-infectious diseases but developed infection few days later during treatment most likely nosocomial infection.

Every patient received more than one antibiotic before culture susceptibility reporting. The most prescribed drug combination was *Cefotaxime* and *Amikacin*, given to 77(90.6%) cases. Combinations of *Vancomycin/Meropenem* (n=3, 3.5%), *Meropenem/Linezolid* (n=3, 3.5%) and *Cefotaxime/Ampicillin* (n=1, 1.2%) were also tried empirically. Only one patient (1.2%) received triple-drug empirical therapy consisting of *Cefotaxime, Amikacin*, and *Meropenem* before culture results.

Overall, antibiotics were prescribed within recommended dosage in 70.2% of cases. In 21.6% of cases, the dosage of antibiotics was less than the optimum dose, while in 8.2% cases, more than the required dose was given (Table-I).

**Table I:** Empirical Antibiotic Therapy – Dosage x Frequency of Prescription.

Empirical Antibiotic Therapy	Recommended Dosage	Less than Optimum Dose	More than Required Dose
Cefotaxime	67%	18%	8%
Amikacin	64%	22%	6%
Meropenem	5%	2%	1%
Vancomycin	2%	0%	1%
Linezolid	4%	0%	0%
Ampicillin	0%	1%	0%

The mean reporting time for culture susceptibility was 4.75 days (SD±1.68). The majority of patients (n=69, 81.2%) received the report within five days of blood sampling. For 16(18.8%) remaining patients, reporting of results took more than five days. One patient had to wait ten days before results were obtained. Gram-negative bacilli were isolated in 69(81.2%) cases. 14(16.5%) cultures were positive for Gram-positive cocci, while *Candida* was found in only two (2.4%) culture susceptibility reports. Organisms isolated are enlisted in Table-II.

**Table II:** Frequency of organisms isolated.

Category	Organism	Frequency
Gram Negative Rods	Pseudomonas	37(43.5%)
	Klebsiella	14(16.5%)
	Serratia S	7(8.2%)
	Acinetobacter	5(5.9%)
	Enterobacter	3(3.5%)
	Escherichia coli	1(1.2%)
	Proteus	1(1.2%)
	Salmonella	1(1.2%)
Gram Positive Rods	Staphylococcus aureus	6(7.1%)
	Coagulase Negative Staphylococcus	5(5.9%)
	Streptococcus	3(3.5%)
Fungi	Candida	2(2.4%)

Total		85(100%)

Gram negative organisms were most susceptible to *Imipenem* (54%), *Piperacillin-Tazobactam* (48%) and *Gentamicin* (48%). Maximum gram-negative samples showed resistance to *Ciprofloxacin* (58%) and *Amikacin* (56%). As a group Gram-positive organisms showed the most susceptibility to *Vancomycin* (100%), *Amikacin* (92%), and *Co-amoxiclav* (85%). Gram-positive organisms were most resistant to *Ciprofloxacin* (91%), *Erythromycin* (79%), and *Co-trimoxazole* (75%). Overall Antibiotic Susceptibility is shown in Table – III.

**Table III:** Collective antibiotic susceptibility of organisms isolated.

Antibiotic	Sensitivity	Resistance	Not Tested
*Imipenem*	54%	40%	6%
*Amikacin*	47%	42%	11%
*Gentamicin*	45%	42%	13%
*Piperacillin/Tazobactam*	38%	39%	24%
*Ciprofloxacin*	33%	56%	11%
*Meropenem*	26%	21%	53%
*Colistin*	22%	0%	78%
*Vancomycin*	16%	0%	84%
*Co-amoxiclav*	15%	19%	66%
*Cotrimoxazole*	11%	26%	63%
*Cefotaxime*	5%	28%	67%
*Linezolid*	5%	0%	95%
*Erythromycin*	4%	14%	82%
*Ampicillin*	2%	17%	81%

Empirical therapy was changed in 27% cases considering the culture susceptibility reporting. In 21% cases, it was changed even before the culture susceptibility report was provided. The change in empirical therapy was delayed by few days even when the pathology department provided the report in 15% cases. In 36.5% cases, no changes in treatment regime were made even after test results.

In most cases (n= 28, 32.9%) treatment was changed to give an antibiotic that was either resistant or not tested during culture susceptibility. There were 22(25.9%) cases in which treatment was changed to an appropriate antibiotic after culture susceptibility testing. Empirical antibiotics were continued in 10(11.8%) cases where results showed that the isolated organism was sensitive to the empirical regimen. However, empirical treatment was continued even after results showed resistance in 5(5.9%) patients. Fourteen(16.5%) patients expired while 6(7.1%) neonates were discharged before their results were available (Table-IV).

**Table IV:** Change in Empirical Therapy: Changed antibiotic x Clinical Outcome.

Alternate Treatment	Clinical Outcome					Total

	Discharged	LAMA	Expired	DOR	Refer	
Changed to Appropriate	7	1	13	0	1	22
Changed to Inappropriate	13	0	12	1	0	26
Continued Appropriate	10	0	0	0	0	10
Continued Inappropriate	5	0	0	0	0	5
Discharged Before Report	5	0	0	1	0	6
Expired Before Report	0	0	14	0	0	14

Total	40	1	39	2	1	83

*Change in Empirical Therapy: Time Taken x Clinical Outcome*						

Before C/S Report	6	1	7	1	1	16
With C/S Report	9	0	13	1	0	23
After C/S Report	7	0	6	0	0	13
Not Changed	18	0	13	0	0	31

Total	40	1	39	2	1	83

**Fig.1:**
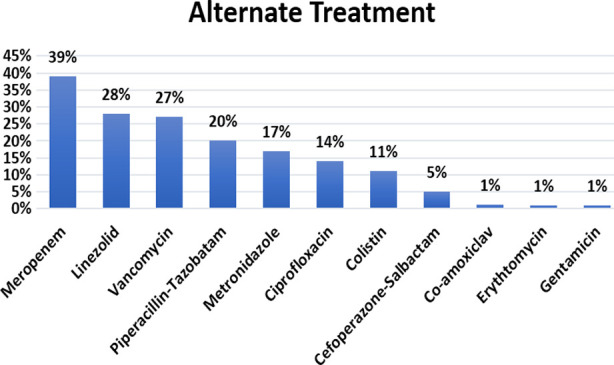
Alternate antibiotic treatment.

*Meropenem* (39%), *Linezolid* (28%), and *Vancomycin* (27%) were the most commonly given substitute antibiotics (Fig.1). After the report patients received combinations of different antibiotics. 16% had monotherapy, 25% each had dual or triple antibiotic regimen, and 22% were given a combination of four antibiotics while 12% received five or more antibiotics after culture results. Antibiotics were changed multiple times after results without any laboratory-proven susceptibility.

The mean duration of indoor stay was 11.35 days (SD±10.68). The minimum stay was of one day for three patients, all of whom expired. One of the patients remained admitted for 50 days until discharged after recovery. Overall, 41 (48.2%) neonates were discharged after recovery, 40 (47%) expired, two (2.4%) discharged on request while, one (1.2%) case was referred to higher specialized hospitals and left against medical advice each.

Most of the death occurred during the first week of hospital stay. It was consistent with the finding that most of these patients had their empirical treatment altered before the culture sensitivity result or were not altered at all. Among the patients who were discharged after recovery most were sent home during the second week of admission; these were the same patients who had their antibiotics altered after considering the culture sensitivity reporting (Table-IV). All the patients (n=10, 11.8%) whose culture sensitivity reporting showed susceptibility to empirical therapy survived (Table-IV).

## DISCUSSION

The neonatal mortality rate is high in developing countries like Pakistan. It is mainly attributed to neonatal sepsis. Major risk factors for neonatal sepsis in our study were LBW, preterm, and birth asphyxia. LBW as a risk factor was noted in many studies,^7^ was found in more than 60% subjects. However, a study by Joshi et al. depicted prematurity as the most common risk factor.^8^ While most recent meta-analysis depicts that LBW is the most common predisposing factor. Most of the patients presented with EOS on the first day of life. Major sources of infection were meningitis and pneumonia. A total of 10 (11.8%) patients were also diagnosed with the nosocomial infection after five days of admission.

Empirical antibiotics were started at first contact with the physician through the intravenous route. The most common prescribed empirical treatment was Cefotaxime/ Amikacin, similar to empirical treatment in Neonatal sepsis in a study conducted in Bahawal Victoria Hospital by Atif et al.^9^ These were the first line of antibiotic treatment according to hospital protocol. However, eight patients also received antibiotics other than this combination. There were 30% of cases where antibiotics were not prescribed within recommended dosage resulting in poor outcomes. The culture susceptibility reporting process was very slow in some cases taking more than 72 hours in most cases leading to a delay in the commencement of appropriate antibiotics. Gram-negative were most commonly isolated organisms, as seen in different national as well as international studies,^3^,^6^,^9^-^12^ and *Pseudomonas* and *Klebsiella* making more than 50% of isolates.^7^ The most common Gram-positive was *Staphylococcus aureus*. Causative bacteria were the same as previous studies but differing in frequency as Klebsiella and Staphylococcus aureus was the most common pathogen in most studies.^3^,^8^,^13^,^14^

In our study, Gram-negative organisms were most susceptible to *Imipenem* (54%), *Piperacillin-Tazobactam* (48%), and *Gentamicin* (48%). High efficacy of *Imipenem* in neonatal sepsis was also established by Obaid Ullah et.al in a study conducted in Peshawar in 2016.^15^. Maximum gram-negative samples showed resistance to *Ciprofloxacin* (58%), *Amikacin* (56%), *Gentamicin* (52%), and *Piperacillin-Tazobactam* (52%) which was the exact opposite of findings in Indian study.^3^ As a group Gram-positive organisms showed the most susceptibility to *Vancomycin* (100%), *Amikacin* (92%), and *Co-amoxiclav* (85%). The gram-positive group was most resistant to *Ciprofloxacin* (91%), *Erythromycin* (79%), and *Co-trimoxazole* (75%).

Cefotaxime, despite being the most prescribed empirical antibiotic was not tested in 57(67%) samples. It was reported sensitive in only four samples (5%), while it was found resistant in 24(28%) cases. Other empirically used drugs Vancomycin, Linezolid, and Ampicillin were also least tested. This could be due to the fact that proper clinical data consisting of clinical assessment and empirical treatment was not provided to the pathology department with a culture request form.

After the results, Meropenem (39%), Linezolid (28%), and Vancomycin (27%) were the most commonly prescribed alternate antibiotics, despite the fact that both of the latter two were least tested during culture sensitivity. Patients received combinations of different antibiotics ranging up to seven antibiotics in a single patient. In many cases treatment was switched to give antibiotics that were not found effective in culture susceptibility testing. The main reasons could be the non-availability of appropriate antibiotics, high cost, or just general disregard for scientific evidence and prioritizing opinion based on clinical experience. This resulted in poor clinical outcomes leading to death of 47% of patients. High mortality rate could be due to the presence of multiple poor prognostic factors like LBW, prematurity, and birth asphyxia as well as poor clinical management ranging from inappropriate dosage, delay in getting test results, delay in starting a specific treatment, or not switching to culture-based treatment at all. A high incidence of Pseudomonas also suggests a probability of nosocomial infection.

This study demonstrated the inconsistencies in the use of antibiotics in neonatal sepsis which yielded in a poor clinical response. It is mainly attributed to lack of latest data available about antibacterial resistance pattern and lack of knowledge of pediatricians about latest antibiotic prescribing protocols. Study also provides recent data about resistance and susceptibility of antimicrobial drugs against different bacteria.

It is high time to develop Antibiotic Stewardship Program considering local sensitivity patterns to prevent the misuse of antibiotics. A multidisciplinary approach should be used to ensure inter-departmental communication so that empirical antibiotics are also tested for susceptibility. Moreover, Microbiologists should help devise the best empirical therapy considering local hospital sensitivity patterns.

### Strength and limitations of the study

Strict follow-up of patients from admission to discharge with proper documentation of reports, empirical as well as specific treatment and clinical outcome is the main strength of this study. However, limitations include patients admitted in indoor ward, and patients treated in outpatients setting were not included in this study. Another limitation is that the data only consist of neonatal patients and lack the information about pediatric patients of different age groups.

## CONCLUSION

Neonatal sepsis is a leading cause of mortality in developing countries. Due to poor availability of latest data about local antibacterial resistance pattern and lack of knowledge among pediatricians about latest antibiotic prescribing protocols, many inconsistencies were noted in the use of antibiotics in neonatal sepsis which resulted in a poor outcome hence, reflecting in international key health indicators (neonatal mortality rate) of country. Optimum management with appropriate use of antibiotics with correct doses will prevent many neonatal deaths due to infective causes. Concerning the change in the resistance pattern of microorganisms to antimicrobials, it is high time to collect local data about antibiotic susceptibility and develop an antibiotic stewardship program to cease inappropriate use of antibiotics.

### Author’s Contributions:

**AH, AU:** Conceptualization of the article.

**AH:** Formal analysis of data.

**MZK, MZK:** Materials and Methodology.

**MZK:** Drafting and manuscript organization.

**MZK, AH:** Review & editing of manuscript.

**AH:** Responsible for the accuracy & integrity of data.
